# Complement as a Biological Tool to Control Tumor Growth

**DOI:** 10.3389/fimmu.2018.02203

**Published:** 2018-09-25

**Authors:** Paolo Macor, Sara Capolla, Francesco Tedesco

**Affiliations:** ^1^Department of Life Sciences, University of Trieste, Trieste, Italy; ^2^Immunorheumatology Research Laboratory, Istituto Auxologico Italiano, IRCCS, Milan, Italy

**Keywords:** complement system activation, tumor control, antibody-based immunotherapy, combination therapies, antibody

## Abstract

Deposits of complement components have been documented in several human tumors suggesting a potential involvement of the complement system in tumor immune surveillance. *In vitro* and *in vivo* studies have revealed a double role played by this system in tumor progression. Complement activation in the cancer microenvironment has been shown to promote cancer growth through the release of the chemotactic peptide C5a recruiting myeloid suppressor cells. There is also evidence that tumor progression can be controlled by complement activated on the surface of cancer cells through one of the three pathways of complement activation. The aim of this review is to discuss the protective role of complement in cancer with special focus on the beneficial effect of complement-fixing antibodies that are efficient activators of the classical pathway and contribute to inhibit tumor expansion as a result of MAC-mediated cancer cell killing and complement-mediated inflammatory process. Cancer cells are heterogeneous in their susceptibility to complement-induced killing that generally depends on stable and relatively high expression of the antigen and the ability of therapeutic antibodies to activate complement. A new generation of monoclonal antibodies are being developed with structural modification leading to hexamer formation and enhanced complement activation. An important progress in cancer immunotherapy has been made with the generation of bispecific antibodies targeting tumor antigens and able to neutralize complement regulators overexpressed on cancer cells. A great effort is being devoted to implementing combined therapy of traditional approaches based on surgery, chemotherapy and radiotherapy and complement-fixing therapeutic antibodies. An effective control of tumor growth by complement is likely to be obtained on residual cancer cells following conventional therapy to reduce the tumor mass, prevent recurrences and avoid disabilities.

## Introduction

Cancer development is a complex biological process that starts with the malignant transformation of normal cells caused by genetic alterations and somatic mutations leading to unrestricted cell proliferation (1). The local microenvironment plays an important role in this process providing favorable conditions for the seeding of cancer cells in a protective niche that allows the growth and expansion of the tumor mass (2). Changes in the structural and organizational properties of extracellular matrix favor adhesion and migration of cancer cells from the initial tumor site (3). Active angiogenesis equally contributes to these environmental changes with the formation of new leaky vessels that supply growing cancer cells with nutrients and promote their metastatic spread to distant organs (4, 5).

Tumor development is constantly controlled by the immune system that recognizes cancer cells as potential threats to body homeostasis and mounts a response leading to local recruitment of effector cells of both innate and adaptive immunity (6). Although cell-mediated immunity has long been recognized to play a critical role in tumor eradication through the action of natural killer cells and cytotoxic T lymphocytes (7, 8), studies reported in recent years have shown that the complement (C) system is also an important player in cancer immune surveillance and these studies have revealed the complex interaction of C with cancer cells. C components are synthesized by resident and recruited cells including fibroblasts, endothelial cells, tissue specific cells, and macrophages (9, 10) and are released in the tumor microenvironment. Biologically active products generated as a result of C activation may directly kill cancer cells or favor their eradication by promoting an inflammatory process. However, it is important to emphasize that C does not always provide an effective protection against tumor growth since its damaging effect on cancer cells can be prevented by C regulatory proteins (CRPs) over-expressed on the cell surface or by other mechanisms of cell resistance to C attack. These evasion strategies are more likely to be operating under conditions of fast tumor growth.

Recent studies have elucidated a novel aspect of C interaction with cancer cells showing that it is able to promote rather than to inhibit tumor development. Markiewski et al. (11) made the original observation that C5a, released in the microenvironment as a result of C activation, recruits and activates myeloid derived suppressor cells that suppress anti-tumor T-cell responses against HPV-induced cancer. Similar findings have been reported in other syngeneic models of mouse tumors invariably associated with C activation (12, 13). Importantly, C5aR1-deficiency and pharmacological blockade of C5aR1 by selective C5aR1 antagonists have been shown to impair tumor growth, pointing to the C5a/C5aR1 signaling axis as an effector mechanism of C-mediated tumor-promoting functions (14).

More recently, C1q secreted in the tumor microenvironment was reported to favor tumor progression by enhancing adhesion, proliferation, and migration of cancer cells and promoting angiogenesis independently on C activation (15).

Given these restraints in C-dependent tumor control, the system has apparently limited chances to provide an effective defense barrier against cancer development unless the C protective functions are made more efficient by optimizing the conditions of its activation and effector activities. In this review, we shall discuss the strategies that may turn the C system into a more efficient therapeutic tool by enhancing its activation on the surface of cancer cells and overwhelming the mechanisms adopted by tumor cells to evade C attack.

## Complement activation at tumor site

Immunohistochemical analysis of tumor tissue has provided useful information on the contribution of the C system to the immune response to cancer revealing the presence of C components in several solid tumors of different tissues and organs. Various cell types in tumor tissue including cancer cells represent the main source of these components which may also derive, at least in part, from the circulation as a result of the increased permeability of the tumor vessels. C deposits have been observed in a number of tumors (16) and in one study that examined the deposition of various C components in glioblastoma, C1q was found to be the most highly expressed component (17). We have recently shown that C1q is present in various tumors in the absence of other C components and exerts functions unrelated to C activation (15). However, this does not exclude C activation at tumor sites as suggested by tissue deposition of known markers of C activation including C4d, C3d, and SC5b-9 (18, 19). Local changes in tumor tissue due to necrosis and apoptosis and, more importantly, inflammation are responsible for C activation to a degree related to the extent of these changes, in particular of the inflammatory process. This suggests that C deposits are likely to be negligible in the initial stages of tumor growth when the inflammatory reaction is hardly detectable and is probably more evident at a later stage of tumor expansion associated with an overt inflammatory reaction. Tumor cells may partly contribute to C activation using cell-bound proteases exposed on their surface to cleave C5 and to generate C5a, which in turn enhances cancer cell invasion (20).

It is not easy to evaluate the impact of C activation at tissue sites on tumor development because the immunohistochemical data have mostly been obtained from well-established cancers. Importantly, C activation products are mainly localized in the tumor microenvironment and found to be weakly or moderately bound to some but not all cancer cells, suggesting that they have limited effect in reducing cell survival. However, it is possible that C exerts a protective effect in the early phase of cancer growth, contributing to induce tumor regression, although this is difficult to ascertain in patients. One way to address this issue is to utilize mice that develop spontaneous tumors and analyze the effect of C activation on its progression at tumor sites. Using BALB/c females expressing the activated rat Her2/neu oncogene, Bandini et al. (21) have shown that the mammary carcinoma developing in C3^−/−^ mice manifests faster growth rate and earlier lung metastasis than the tumor in wild type animals, suggesting that C activated by antibodies (Abs) directed against Her2/neu oncogene and/or other tumor-associated antigens may control tumor growth. Different results were obtained using a syngeneic mouse model of ovarian cancer which showed similar growth in wild type and C3^−/−^ mice due to secretion of C3 by tumor cells that exerts a stimulating effect on cell proliferation (22). Overall, the available data support a dual role of C in tumor immune surveillance and its ability to either prevent or promote tumor progression depends on the characteristics of cancer cells and the anti-tumor efficiency of the C system.

## Cancer cells as potential target of complement attack

Expression of tumor-associated molecules on cells undergoing malignant transformation can lead to C activation on the cell surface by all three activation pathways. The lectin pathway has been implicated in C activation on glioma cells which express, like many other malignant cells, high mannose glycopeptides that bind MBL and trigger consumption of C4 and C3, but this reaction fails to induce cell lysis (23). Virus transformed cells express novel antigens that are able to activate the alternative pathway, as is the case of EBV-infected B lymphoblastic cell lines (24–26) and T and monocytic cell lines infected by HIV (27). The classical pathway of C can be activated on cancer cells by natural Abs, preferentially of IgM isotype, that recognize carbohydrate moieties on cell surfaces (28, 29). Cytotoxic Abs reacting with carbohydrate epitopes of gangliosides GD2 and GD3 on neuroblastoma and melanoma cell lines have been detected in a small number of sera from normal individuals (30). Unfortunately, besides the low frequency, the natural Abs are not efficient in promoting C-mediated cell killing due to their low titer and affinity.

Attempts have been made to vaccinate cancer patients with the aim to induce production of therapeutic Abs. The anti-tumor response has not always been satisfactory, although a novel vaccination procedure has recently been developed in rabbits to stimulate the generation of IgG Abs that cause strong C-mediated lysis of myeloma cells carrying the CD38 antigen (31).

Despite this improvement, the development of recombinant Abs against tumor antigens remains the preferential approach to stimulate selective C activation on cancer cells, although the identification of specific tumor-associated antigens able to discriminate cancer cells from healthy tissue still represents a major limitation in Ab-mediated cancer therapy. A major progress has been made in the immunotherapy of hematologic malignancies, in particular those derived from B cells, with the generation of monoclonal Abs directed against target antigens, such as CD19 and CD20, present only on B-cells at late stages of development, and not on hematopoietic stem cells that are therefore unaffected by the treatment. Conversely, the development of therapeutic Abs against solid tumors has been limited by the difficulty to identify specific target antigens on cancer cells, whether overexpressed self-antigens, or neoantigens due to tumor-specific mutations or oncogenic viruses.

Only 15 monoclonal Abs have been approved by FDA for the treatment of all different solid tumors (32), and only 3 of them are C-fixing molecules, as described in Table 1.

**Table 1:** FDA-approved complement-fixing antibodies.

**Target antigen**	**INN**	**Company**	**Source**	**Year of first US FDA (EU EMA) approval**	**Therapeutic indication(s)**
CD20	Ibritumomab tiuxetan	Biogen Idec	Murine IgG1 (type I)	2002 (2004)	NHL
	Ofatumumab	Genmab and GSK	Human IgG1 (type I)	2009 (2010)	CLL
	Rituximab	Biogen Idec, Genentech (Roche)	Chimeric IgG1 (type I)	1997 (1998)	NHL; CLL
CD38	Daratumumab	Janssen-Cilag, Genmab	Human IgG1/κ	2015 (2016)	MM
CD52	Alemtuzumab	Millennium Pharmaceuticals and Genzyme	Humanized IgG1	2001	CLL
EGFR	Cetuximab	ImClone (Eli Lilly), Merck Serono and BMS	Chimeric IgG1	2004	Head and neck cancer; colorectal cancer
GD2	Dinutuximab	United Therapeutics Europe	Human IgG1/κ	2015	Neuroblastoma
HER2	Pertuzumab	Roche	Humanized IgG1	2012 (2013)	Breast cancer

*INN, International Non-proprietary Name; NHL, Non Hodgkin Lymphoma; CLL, Chronic Lymphocytic Leukemia; MM, Multiple Myeloma*.

## Factors affecting the efficiency of the antibodies to activate complement: the antibodies structure

Among the molecular characteristics of recombinant Abs responsible for C activation, the Ig class is critically important since human IgM, IgG1, and IgG3 are known to be the most effective C activators whereas IgG4 fails to bind C1q (33). The structure of the Fc region of these Abs has been extensively investigated to improve their therapeutic efficiency and changes of some amino acids in this region were found to enhance the Ab activity (34). In particular, computational design followed by high-throughput screening techniques has allowed the identification, production, and characterization of Fc variants with increased ability to bind C1q and to promote C-dependent cytotoxicity (CDC) (35).

Glycosylation is an important secondary modification of immunoglobulins that has a significant impact on their capacity to activate different arms of the immune system. The addition of conserved glycans, in particular α (1,6)-linked core fucose, to the Fc region, was shown to be critical for the interaction of the Ab with the C system (36). This observation has raised strong research interest in several biotech companies, resulting in the commercialization of the anti-CD20 Ab obinutuzumab (Genentech, San Francisco, CA, USA). Terminal mannosylation is another important post-translational modification that prolongs the half-life of the Abs in the circulation and favors binding of mannose-binding lectin (MBL) (36). Importantly, the terminal glycosylation of IgG has been shown to influence CDC without affecting Ab-dependent cell cytotoxicity (ADCC). In addition, an increased content of terminal galactose potentiates CDC activity by enhancing the binding of C1q to the modified Ab (37).

The discovery that hexamerization of Abs after binding to target antigens leads to a successful activation of the classical pathway represents a major advance in the development of new strategies to enhance C activation by IgG (38, 39). The critical role of this process in C activation is supported by the finding that some mutations of Fc amino acid sequence of anti-CD20 IgG result in impaired hexamer formation and reduced cell lysis. Conversely, other mutations have the opposite effect and similar results were obtained introducing the same mutations in the IgG4 isotype. A certain degree of flexibility of antigen-bound Abs allows a conformational change required for hexamerization. The ability of anti-CD20 Abs to exhibit a more efficient CDC after hexamer formation is shared also by anti-CD52 and anti-HLA Abs (38, 39).

## Factors affecting the efficiency of the antibodies to activate complement: the antigen

Irrespective of the C-activating capacity of anti-tumor Abs, the characteristics of the target antigen remain of pivotal importance for a successful tumor cell lysis. The beneficial effects of Abs in cancer immunotherapy depend on the expression pattern and the tissue specificity of tumor antigen that should be present exclusively or predominantly on cancer cells to allow selective or almost exclusive targeting of tumor cells. It is equally important that the tumor antigens are expressed also on metastatic cells which represent the main target of Ab-based immunotherapy since other therapeutic approaches including surgery, radiotherapy, and chemotherapy can be used to obtain an effective control of primary tumor. Moreover, the tumor antigens should be stably expressed on the cell surface to serve as useful targets for immunotherapy, whereas intracellular antigens, though specific for tumor cells, can only be used for diagnostic purposes. It is important to point out that tumor cells are often heterogeneous in the expression level of tumor antigens that may influence their susceptibility to CDC. We have observed that cells expressing low levels of CD20 isolated by cell sorting from a population of either chronic lymphocytic leukemia (CLL) or cancer B-cell lines and kept in culture for over a week give rise to cells expressing higher level of CD20 that more easily undergo C-mediated lysis (40, 41). This observation suggests that repeated injections of Abs administered at appropriate time intervals can be used to allow the emergence of cell clones expressing higher levels of CD20 and more susceptible to CDC. Finally, the release of antigen in the tumor microenvironment and in the circulation may lead to blockade of therapeutic Ab and contributes to reduce its expression level on the cell surface, making cancer cells less susceptible to Ab-mediated C-dependent killing (9).

The number of antigenic sites does not always account for the capacity of a monoclonal Ab to cause CDC. In this regard, the impact of the different distribution of two tumor antigens, the alpha isoform of folate receptor (42) and CD20 (43), on Ab binding and C activation has been compared. The folate receptor is associated with epithelial ovarian carcinoma cells and is expressed on several cell lines at a concentration of about 1 × 10^6^ molecules/cells (44). CD20 is present on cancer B-cells at a substantially lower expression level of around 40,000–70,000 molecules/cell (45). Despite the marked difference in the number of cell-associated antigenic sites, the chimeric anti-CD20 Rituximab is able to activate C (46) and to kill B cells whereas a chimeric anti-folate receptor Ab fails to do so (42).

Additional factors may play a relevant role in promoting a more efficient C activation by recombinant Abs, including the proximity of the target epitopes to the cell surface (47), the density of target antigen (48), and the Ab-induced movement of the antigens across the cell membrane (49).

A recent study by Cleary and colleagues provided convincing evidences that the efficacy of C-mediated killing of cancer cells induced by Ab is largely influenced by the distance of the target epitope from the cell membrane and the greater the distance from the cell surface, the lower the efficiency of cell lysis (47). They used target cells transfected with fusion proteins containing either CD20 or CD52 epitopes attached to various CD137 scaffolds and showed that the cells displaying the target epitopes closer to the membrane were more susceptible to CDC than those expressing the epitopes furthest away from the cell surface. These data clearly suggest that the position of the epitope in the target antigen is an important factor to consider in the selection of therapeutic Abs. The surface expression level of the antigen has been shown to be equally important for an efficient C activation on both hematological and solid tumors. Golay et al. (45) analyzed freshly isolated cells from patients with B-CLL and prolymphocytic leukemia for CDC induced by Rituximab and found that the C sensitivity of these cells correlated with the surface expression of CD20. Derer et al. (48) reached similar conclusions using a fibroblast cell line expressing different levels of Epithelial Growth Factor Receptor (EGFR) and reported data indicating that the cell susceptibility to CDC progressively increased at higher expression level of EGFR. An increased antigen density resulting from Ab-induced movement of the tumor-associated antigen across the cell membrane can also contribute to enhance Ab-dependent C activation. CD20 is an example of a membrane antigen, that is induced by the type 1 Abs rituximab and ofatumumab to translocate to the lipid rafts (49). As a consequence, the immune complexes reach a critical concentration required for hexamer formation and C1q binding (50).

## Factors affecting the efficiency of the antibodies to activate complement: the complement system

C is an important player in Ab-induced tumor cell death and has therefore a major impact on the efficacy of therapeutic Abs. A clinical observation in patients with CLL treated with Abs is that the depletion of cancer and normal cells in the blood of patients is impaired in the presence of reduced levels of C components (51). Clearance of CLL cells induced by Abs to CD20 has been shown to be associated with C consumption, particularly of the early components, which persist for several days to weeks (52). This would cause a reduced therapeutic effect of subsequent infusions of the same Abs to control the malignant cells that circulate in blood in increasing number due to migration from bone marrow or lymph node. Using an *in vitro* model to evaluate the CDC of Burkitt's lymphoma cell lines induced by ofatumumab and rituximab, Beurskens et al. (51) have investigated the effect of different concentrations of anti-CD20 Abs on cell killing in two consecutive steps. They found that the dose of anti-CD20 Abs tested in the first step was critical for the degree of cell killing in the second step. In particular, using the maximal dose of anti-CD20 Abs in the first step, the cell lysis did not exceed 30% in the second step, while the percentage of cell killing increased to over 80% using a lower Ab concentration in the first step. These data suggest that the best therapeutic option would be to use the minimal concentration of Ab to trigger C-mediated killing of a relatively high number of cells leaving a C level sufficient to clear newly emerging malignant cells treated with an additional administration of Ab.

The critical role of C in CDC induced by recombinant Abs is supported by other uncontrolled studies suggesting that the killing of cancer B cells could be enhanced based on supplementation with purified C components or fresh frozen plasma (53, 54).

The response to immunotherapy of tumors that develop extravascularly is likely to be different from that of circulating cells. Unfortunately, it is difficult to evaluate the concentration of the Ab at cancer site, nor is it easy to measure the activity of the C system in the tumor microenvironment. However, the amount of Abs that reaches tumor sites (55) should be sufficient to activate C if the Abs tend to form hexamers that require limited amount of C components to activate the system (39).

Evidence supporting local C deposition was obtained by our group using a mouse xenograft model of B-cell lymphoma established in SCID mice with the intraperitoneal injection of a lymphoma cell line (56). This model is characterized by the development of peritoneal tumor masses and formation of foci of lymphoid cells in the spleen, liver, and bone marrow. Injection of rituximab into tumor-bearing mice resulted in the deposition of the Ab, C3, and C9 on tumor cells and in prolonged survival of these animals.

## Complement-mediated cancer cell damage and regulation

The importance of late C components in tumor development has recently been investigated by Verma et al. (57) in a xenogenic mouse model of B-cell lymphoma. They showed that tumor-bearing C5 deficient animals treated with rituximab died within the 52 days period of observation whereas all C5 sufficient mice survived. Although the tumor tissue was not examined for complement deposition, the membrane attack complex (MAC) is likely to have contributed to the C protective effect in this model.

MAC assembly on the cell membrane is the final step of C activation. Tumor cell killing caused by Ab-mediated C activation takes a few minutes to complete under standard *in vitro* conditions (52) and is largely mediated by increased Ca^2+^ influx and rapid activation of a large variety of enzymes as a result of MAC insertion (58, 59). C5a and other C activation products can also contribute to tumor control by recruiting to the tumor microenvironment inflammatory cells that cause cell death via C-dependent cell cytotoxicity and phagocytosis (60).

A large body of evidence has been collected showing that cancer cells can resist CDC by several different mechanisms acting either on the cell surface or intracellularly.

Removal of MAC from the cell surface is one of these mechanisms observed in different tumor cell types after the activation of the C system by mAbs (61–64). This removal is usually mediated through membrane vesiculation, directed both to the inner and the outer sides of cell surface (65).

Overexpression of the membrane-associated C regulatory proteins (mCRPs) CD46, CD55, and CD59 is another mechanism by which cancer cells can evade undesired C attack due to spontaneous or Ab-induced C activation. The mCRPs act at different steps of the C sequence by favoring the decay of the C3 convertases (CD55), promoting the degradation of C3b and C4b (CD46), and preventing the assembly of MAC (CD59) (66, 67). Because of their high expression level on several tumors, mCRPs are considered promising targets for cancer immunotherapy. CD46 has been shown to be highly expressed on colorectal, breast (68), prostate, lung, liver, and ovarian carcinoma (69) cancer cells. Elevated levels of CD55 have been documented in a wide range of cancers including lung, colorectal, gastric, breast, and cervical cancers as well as in leukemia (66). CD59 is also overexpressed on different types of carcinoma and sarcoma and on melanoma cells (70).

An important point to emphasize is that hyper-expression of mCRPs on the surface of tumor cells does not necessarily mean that they are equally involved in cell protection from C attack. Almost two decades ago, we analyzed various B-lymphoma cell lines for their susceptibility to CDC and found that all expressed increased levels of CD55, CD46, and CD59 and were variably resistant to C lysis (46). However, using neutralizing Abs to mCRPs, we were able to show that the resistance to C-dependent cell lysis was abrogated by blocking the inhibitory activity of CD55 and CD59 whereas inhibition of CD46 was totally ineffective (46). In contrast, CD46 appears to play a more prominent role in protecting ovarian cell lines from C attack as suggested from the substantial increase in C-mediated cell lysis observed inhibiting CD46 activity with anti-CD46 neutralizing Abs (42). These findings have important clinical implication for the selection of mCRP to inhibit in the immunotherapy of different tumors.

## Therapeutic strategies

### Antibodies and complement activation

Over the past 20 years, therapeutic Abs have rapidly become the leading product in the biopharmaceutical market. Currently, there are more than 30 FDA-approved therapeutic Abs for cancer treatment and some of them are C-fixing Abs that mediate CDC (Table 1).

Rituximab was the first C-fixing Ab to receive FDA approval and has been used successfully to treat a large number of patients with CD20-expressing B-cell malignancies. Because CD20 is expressed on several B cell-derived cancer cells and also on normal cells from the late pro-B cell through memory cells, while absent on plasma cells and precursors hematopoietic stem cells, it is understandable why treatment with anti-CD20 Abs induces depletion of cancer cells but does not interfere with the repopulation of the B-cell compartment (71). Analysis of the binding mode of anti-CD20 Abs and their epitope specificity has led to the identification of two types of Abs that differ in their ability to form distinct complexes with CD20. Type I Abs stabilize CD20 in lipid rafts leading to stronger C1q binding and increased C activation whereas type II Abs exhibit reduced C1q binding that results in lower levels of cell death mediated by CDC (72).

Rituximab, ofatumumab and ibritumomab tiuxetan are examples of type I Abs known to be efficient activator of the C cascade (71). On the contrary, type II Abs like tositumumab performed poorly in CDC (49) and the same was observed for the optimized type II Ab obinutuzumab which fails to induce CDC (72, 73). However, Bologna et al. have reported that C plays a role in cell killing induced by high dose of the type II glycoengineered anti-CD20 mAb obinutuzumab on B-CLL expressing high levels of CD20, as suggested by the ability of the anti-C5 Ab eculizumab to totally prevent cell lysis (74).

In addition to anti-CD20 Abs, other approved C-fixing Abs directed against various tumor antigens have been recognized to activate C both *in vitro* and *in vivo* including anti-CD52 alemtuzumab (75), anti-CD38 daratumumab (76, 77), anti-EGFR cetuximab (78, 79), anti-GD2 dinutuximab (80) and anti-HER2 pertuzumab (81).

### Strategies to improve complement activation by therapeutic antibodies

#### Combination of different antibodies

The efficiency of C activation on the cell surface is largely influenced by the epitope density of the antigen recognized by recombinant Abs which can affect the formation of an adequate number of immune complexes capable of binding C1q. Two different strategies have been reported to increase the formation of C1q-fixing dimers. One approach is to use a combination of two Abs recognizing different epitopes on the same antigen sufficiently close to allow juxtaposition of the IgG Abs which is critical for C1q binding. Spiridon et al. (82) were the first to analyze C-mediated lysis of Her-2^+^ human breast cancer cell lines induced by several mAbs and showed an enhanced killing using a mixture rather than individual mAbs. Our group has investigated the C-fixing ability of two Abs, cMOV18, and cMOV19, that bind to distinct epitopes of the alpha isoform of folate receptor, highly expressed on epithelial ovarian cancer cells. Interestingly, the mixture of these two Abs was able to activate C and to cause death of ovarian cancer cells while individual Abs were totally ineffective (42). A similar pattern of C activation was obtained using combination of the anti-EGFR Abs cetuximab and matuzumab, which recognize different nonoverlapping epitopes of EGFR (79). Cetuximab was reported to induce some degree of CDC in lung cancer cell lines only at high concentrations (40 μg/mL) (79), whereas lower amount (10 μg/mL) of either cetuximab or matuzumab failed to trigger C activation. Interestingly, the mixture of the two Abs was able to induce C1q and C4c fixation leading to strong activation of CDC (50 and 80% of lysis of squamous cell carcinoma and glioblastoma cells, respectively) (78).

Although this approach has not yet been introduced in clinical practice, it represents a promising future development in immunotherapy with C-fixing Abs.

#### Neutralization of membrane complement regulatory proteins

Different approaches based on anti-mCRP Abs or silencing mCRP expression in combination with therapeutic Abs have been evaluated *in vitro* and *in vivo* by various groups to prevent the C-inhibitory effect of mCRP. We initially reported an increased susceptibility of follicular and Burkitt's lymphoma cell lines to CDC induced by Rituximab in the presence of Abs to CD55 and CD59 (46). These findings were later confirmed using an *in vivo* model of human CD20+ B-lymphoma established in severe combined immunodeficient mice treated with rituximab in combination with anti-CD55 and anti-CD59 Abs that resulted in a significant animal survival (56). Similar enhancing effect of anti-CD55 and anti-CD59 Abs was reported on C-mediated killing of two human lung carcinoma cell lines induced by Herceptin (trastuzumab) (83). Neutralizing Abs to CD46 and CD59 were instead required to enhance CDC of ovarian carcinoma cells induced by the mixture of cMOV18 and cMOV19 (42). Down-regulation of all three mCRPs obtained with cationic liposomes (AtuPLEXes) loaded with siRNAs proved effective in inducing substantial increase of CDC of HER2 positive breast, lung and ovarian adenocarcinoma cell lines stimulated by trastuzumab and pertuzumab (84).

Although lysis of C-resistant tumor cells can be restored by the addition of Abs neutralizing mCRPs, their use is limited by the ubiquitous expression of mCRPs on both normal and tumor cells. One way to avoid undesired side effects that may derived from the binding of Abs to normal cells resulting in decreased expression of mCRPs is to selectively deliver the Abs to tumor cells. To this end, our group has generated two bispecific Abs containing binding specificity to CD20 and either CD55 or CD59. These Abs were able to recognize CD20 expressed on Burkitt's lymphoma cell lines and to neutralize membrane-bound CD55 and CD59 enhancing cell susceptibility to C-mediated lysis. An *in vivo* model of Burkitt's lymphoma developed in SCID mice was used to investigate the tissue distribution of bispecific Abs that were found to target selectively the tumor mass due to the high affinity of the anti-CD20 portion as opposed to the lower affinity of the anti-CD55 or anti-CD59 arms and to prevent tumor development (60). The therapeutic effect of the Abs was largely dependent on C activation, as revealed by the increased deposition of C3 and C9 in tumor masses, and also by local recruitment of macrophages and NK cells. Importantly, the combination of these two bispecific Abs resulted in the survival of 100% of treated mice whereas treatment with a single bispecific recombinant Ab (MB20/55 or MB20/59) induced the survival of only 20% of animals (60).

An interesting approach aimed at inhibiting mortalin, an heat shock protein over-expressed in many cancer types, has been proposed by Fishelson and his group (85) to interfere with MAC formation and its release from cell surface. The level of mortalin is inversely related to MAC deposition and its over-expression in erythroleukemic cells protects from C activation through the classical pathway, while protein down-regulation using specific siRNA increases the level of cell-bound C9.

## Complement activation potentiates standard therapies

### Complement and radiotherapy

Together with surgery and chemotherapy, radiotherapy is a clinical mainstay of treatment for many malignancies especially for aggressive tumors with poor prognosis.

Recent studies support an association between radiation therapy for both human and murine cancers and C activation. An elegant study by Surace et al. (86) showed that local irradiation of melanoma and colon carcinoma developing in mice with a single dose of 20 or 5 Gy resulted in rapid and transient C activation triggered by tumor cells undergoing necrosis, apoptosis, and mitotic catastrophe with the possible contribution of natural IgM Abs bound to necrotic cells. In addition, they documented tumor deposition of C3 activation products and local increases in C3a and C5a, which induce maturation and activation of tumor-associated dendritic cells expressing the receptors for these anaphylatoxins and in turn promoting the anti-tumor activity of CD8^+^ T lymphocytes. The important role of C activation in the control of tumor growth was supported by the finding that radiotherapy failed to exert a protective effect against the tumor in mice deficient in either C3, or in C3a or C5a receptors, suggesting the critical contribution of locally released C3a and C5a.

Somewhat different results were obtained by Elvington et al. (87) who used a lymphoma model in mice receiving low dose radiotherapy fractionated over a period of approximately 5 months. They found that C inhibition induced by the administration of CR2-Crry resulted in longer survival and reduced tumor mass in tumor-bearing mice. A possible explanation for these contrasting results is that a radiation treatment administered over a prolonged period of time induces a C-independent inflammatory response that contributes to promote tumor growth. Overall, these data indicate that dose and fractionation in the radiation therapy need to be further investigated to find optimal conditions that combine the beneficial anti-tumor effects of radiotherapy and C activation.

### Complement and chemotherapy

Limited information is available on the interplay between the C system and chemotherapeutic agents and the effect of this interaction on tumor control.

Levels of C3 and C4 were measured in patients with breast cancer treated with epirubicin/docetaxel-based neoadjuvant chemotherapy and found to be substantially reduced (88). This finding cannot be explained by C consumption because the low concentrations of C3 and C4 were not associated with a parallel increase in the level of the C activation product C4d. The relevance of this observation is unclear since the levels were equally reduced in responders and non-responders to chemotherapy. A similar conclusion was reached in another study that examined the changes in C activity in patients with various types of cancer and revealed a significant reduction in C activity which was not accompanied by a corresponding increase in the level of C3d (89). More direct evidence supporting the beneficial effect of a combination therapy with a chemotherapeutic agent and a recombinant Ab was obtained from a multicenter clinical trial conducted in patients with advanced non-small-cell lung cancer (90). The patients receiving the monoclonal Ab cetuximab directed against epidermal growth factor receptor (EGFR) in combination with cisplatin/vinorelbine survived longer than those treated with the chemotherapeutic agents alone. In a subsequent study, cetuximab was found to bind to a lung cancer cell line expressing EGFR and to activate C resulting in the assembly of the membrane attack complex and cell death. C involvement in the killing of tumor cells was further documented by the finding that the inhibitory effect of cetuximab on tumor growth in an *in vivo* xenogeneic model of A549 lung cancer cells in nude mice was abolished in tumor-bearing mice treated with cobra venom factor to deplete C (79). These are promising results that need to be confirmed using a similar approach in the study of other tumors because the effect of chemotherapy on C activation and the consequent impact of these treatments on cancer cell killing may be different in various tumors.

## Conclusion

The introduction of recombinant Abs into the clinic to control tumor growth has fostered the interest in C as an anti-tumor defense system acting in close collaboration with other components of both innate and acquired immunity. This has prompted the development of various strategies to optimize their therapeutic efficiency including structural modifications of the Abs to promote C activation and also control C inhibitors expressed on the tumor cell surface to enhance Ab-induced C-mediated cell killing. Major efforts are being made to selectively deliver mCRPs neutralizing agents to tumor cells and the recently generated bispecific Abs that target cancer cells and inhibit mCRP appear to move in this direction.

**Figure 1:**
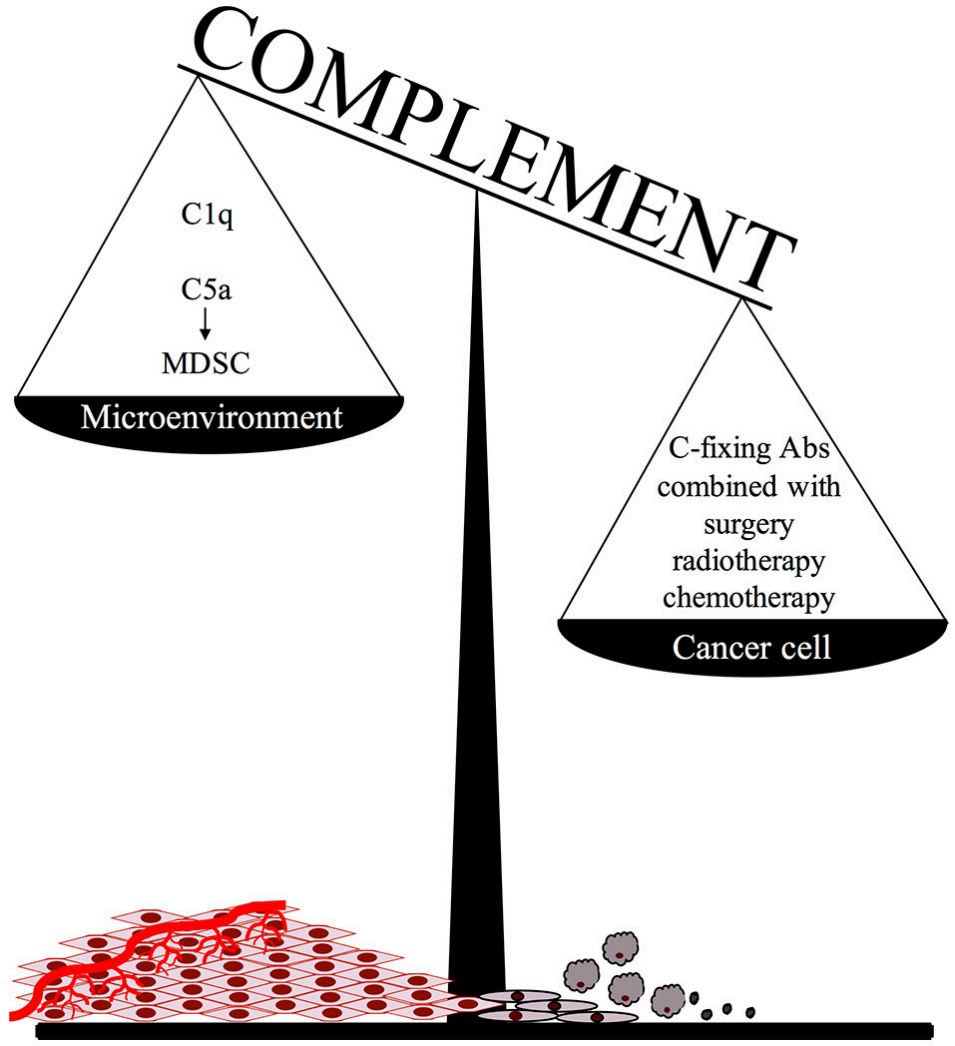
The ambivalent role of complement in tumor growth.

An important point to consider when adopting this therapeutic approach is that C activated in the tumor microenvironment, particularly in the case of slow growing tumors associated with an inflammatory process developing in the surrounding tissue, may promote cancer expansion due to recruitment of suppressor cells by locally released C5a (Figure 1). We believe that this undesired effect may be prevented or markedly reduced by focusing Ab dependent C activation on residual tumor cells after surgical removal or substantial reduction of tumor mass after radio and/or chemotherapy. It is important, though, that the protocols for radiation therapy and chemotherapeutic treatment are selected to be highly effective in the control of tumor growth with limited pro-inflammatory side effects and negligible C activation in the tumor microenvironment.

## Author contributions

All authors listed have made a substantial, direct and intellectual contribution to the work, and approved it for publication.

### Conflict of interest statement

The authors declare that the research was conducted in the absence of any commercial or financial relationships that could be construed as a potential conflict of interest.
